# Health and care system assessment aimed at cultural adaptation of MhGAP modules

**DOI:** 10.1192/j.eurpsy.2023.382

**Published:** 2023-07-19

**Authors:** E. Dozio, L. Caron

**Affiliations:** Action contre la Faim, Paris, France

## Abstract

**Introduction:**

As part of the Global Mental Health (GMH) movement, WHO launched the Mental Health Gap Action Program (mhGAP). One of the key initiatives of the MhGAP is to train lay health professionals to meet the mental health needs of populations, particularly in low- and middle-income countries (LMICs) where the need far exceeds the availability and quality of services. Training modules are standardized and designed for use in many countries and settings. In practice, there is often a stereotyped reproduction of modules without consideration of specific cultural adaptation needs.

**Objectives:**

As part of a psychosocial support program in two provinces of the eastern Democratic Republic of Congo (Ituri and North Kivu), the NGO Action contre la Faim proposed a health and care system assessment aimed at adapting MhGAP modules for health center staff. The objective was to know the local practices in terms of psychic care as well as to identify the competences already existing to reinforce them, but also to contextualize the tools and the contents of the trainings.

**Methods:**

The methodology used was mixed.

A questionnaire based on the WHO situation analysis tools was revised, simplified and adapted to the zone, allowing us to obtain quantitative data on the health centers, the care provided, referrals and supervision. Health care workers were interviewed using the questionnaire from ACF’s “Strengthening the Health Care System” guide, focusing on care methods, knowledge of mental health and the most frequently encountered symptoms. Focus Group Discussions with the Community Relais in the area allowed for the collection of information on the level of knowledge of the population in terms of mental health, their awareness on this subject, their cultural vision and the means of care.

**Results:**

The data collected from 9 health centers in the two provinces allowed us to learn about:

- Poor knowledge of mental health and school readiness

- Identification of barriers to access to care

- Beliefs around mental health, mental suffering and care

- Details about the different pathologies and symptoms as well as the issues related to the therapeutic framework.

**Conclusions:**

The evaluation was fundamental to have a better knowledge of the context, which made it possible to adapt the content of the MhGap training modules les, to design tools more adherent to reality. The data collected may also be the subject of advocacy aimed at mobilizing the country’s policies in terms of mental health, as well as raising awareness in the international community.

**Disclosure of Interest:**

None Declared

